# *Annona cherimola* Seed Extract Activates Extrinsic and Intrinsic Apoptotic Pathways in Leukemic Cells

**DOI:** 10.3390/toxins11090506

**Published:** 2019-08-30

**Authors:** Tony Haykal, Peter Nasr, Mohammad H. Hodroj, Robin I. Taleb, Rita Sarkis, Marvy Nadine El. Moujabber, Sandra Rizk

**Affiliations:** 1 Department of Natural Sciences, Lebanese American University, Beirut 13-5053, Lebanon; 2 Laboratory of Regenerative Hematopoiesis, Swiss Institute for Experimental Cancer Research (ISREC) & Institute of Bioengineering (IBI), School of Life Sciences, Ecole Polytechnique Fédérale de Lausanne (EPFL), 1015 Lausanne, Switzerland; 3 Department of Health Policy and Management, City University of New York, New York, NY 10027, USA

**Keywords:** *Annona cherimola* Mill, apoptosis, cancer, leukemia, toxic

## Abstract

*Annona cherimola* Mill is a large green fruit with black seeds widely known to possess toxic properties due to the presence of Annonaceous acetogenins. The present study investigates the anti-cancer properties of an *Annona cherimola* Mill ethanolic seed extract on Acute Myeloid Leukemia (AML) cell lines in vitro and elucidates the underlying cellular mechanism. The anti-proliferative effects of the extract on various AML cell lines and normal mesenchymal cells (MSCs) were assessed using WST-1 viability reagent. The pro-apoptotic effect of the extract was evaluated using Annexin V/PI staining and Cell Death ELISA. The underlying mechanism was deciphered by analyzing the expression of various proteins using western blots. Treatment with an *A. cherimola* seed ethanolic extract promotes a dose- and time-dependent inhibition of the proliferation of various AML cell lines, but not MSCs. Positive Annexin V staining, as well as DNA fragmentation, confirm an increase in apoptotic cell death by upregulating the expression of pro-apoptotic proteins which control both intrinsic and extrinsic pathways of apoptosis. GC/MS analysis revealed the presence of phytosterols, in addition to other bioactive compounds. In conclusion, *Annona cherimola* Mill seed extract, previously known to possess a potent toxic activity, induces apoptosis in AML cell lines by the activation of both the extrinsic and the intrinsic pathways.

## 1. Introduction

Cherimoya, fruit of the tree of *Annona cherimola* Mill. and member of the custard apple family (Annonaceae), is a large green fruit with round protrusions, white pulpy flesh, and black bean-size seeds embedded in the pulp. *Annona cherimola* Mill. is the official name of the plant, as featured on theplantlist.org [1].

Traditionally, *A. cherimola* extract has been used for various purposes, mostly due to its potent toxic activity [2]. Crushed seeds of cherimoya have been used as an insecticide, and for the treatment of lice and parasitic skin infections [3]. In fact, contact of *A. cherimola* seed extract with the eyes incidentally caused blindness and ingestion of the extract caused gastrointestinal disturbances such as nausea, vomiting, flatulence, and atropine-like effects, including photophobia and dryness of the mouth [3].

Important toxic components of the seeds include the annonaceous acetogenins (ACGs). These are considered environmental neurotoxins responsible for neurodegenerative diseases like atypical Parkinsonism and dementia in areas known for cultivating *A. cherimola* [4]. Moreover, an injection of ACGs in mice increased the proportion of polychromatic erythrocytes, indicating the ACGs’ in vivo genotoxic capacity [5].

Furthermore, close species of the Annona genus have also been shown to possess toxic activity. In fact, *A. squamosa* and *A. muricata* seed extracts have been used as natural insecticides against Madagascan mosquitoes [6]. Additionally, more than 100 ACGs have been isolated from the leaves, barks, seeds, roots, and fruits of *A. muricata* [7]. Some of these ACGs are considered responsible for Guadeloupean atypical Parkinsonism [8]. Other phytochemicals, like alkaloids, extracted from *A. muricata* were shown to be neurotoxic against dopaminergic neurons, which explains the neuronal dysfunction and degeneration underlying the West Indian parkinsonian syndrome [9]. All these studies shed light on the significant risks of neurodegeneration related to the consumption of Annona species fruits.

On the other hand, *A. cherimola* is considered to have some health benefits when included in the diet [10]. Extracts from the seeds possess antioxidant and anti-inflammatory activities [11]. It has even been traditionally considered to have anti-cancer properties according to some accounts in Mexico [12].

Recently, research has investigated the pharmacological activities exhibited by different parts of the plant, notably leaf extracts, which showed cytotoxicity against several microbial species, as well as breast, colon, and liver cancer cell lines [13]. Annonaceous acetogenins extracted from *A. cherimola* seeds exhibited cytotoxic effects against prostate, breast, and colon cancer cell lines, with a 10,000 times stronger potency than adriamycin, an important chemotherapeutic drug [14]. However, these effects were not attributed to the toxins of the extract or to any other pharmacological effect of the extract.

Acute Myeloid Leukemia (AML) is a cancer that affects the blood and bone marrow and it includes a group of leukemia that develops in all blood cells, excluding lymphocytes [15]. Treatment for AML is currently focused on the use of cytotoxic or cytostatic drugs that theoretically target cancer cells rather than healthy body cells. Medicinal plants have historically proven their value as a source of molecules with therapeutic potential, and nowadays represent an important pool for the identification of novel drugs [16], to decrease proliferation and metastasis of cancer, when the plant extract is used either alone [17], or in combination with known chemotherapeutic drugs [18,19].

The present study investigates the anti-cancer properties of the known toxic extract from the seed of *A. cherimola* on AML cell lines in vitro and elucidates its mechanism of action.

## 2. Results

### 2.1. A. cherimola Mill Seeds Extract Selectively Reduces the Proliferation of AML Cell Lines

**Figure 1:**
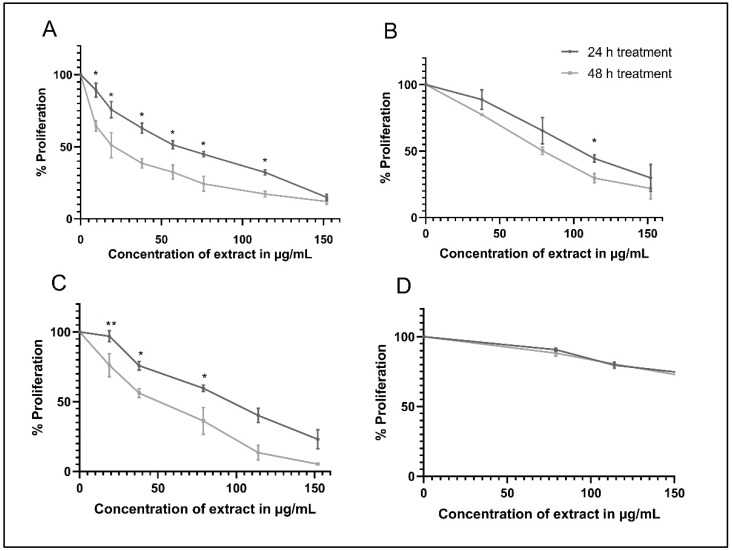
Proliferation of acute myeloid leukemia (AML) cell lines, namely KG-1 (**A**), U937 (**B**), Monomac-1 (**C**), and normal mesenchymal cells (MSCs) (**D**) treated with *Annona cherimola* seed ethanolic extract (ASEE) for 24 h and 48 h. A significant dose- and time-dependent inhibition of AML cell proliferation is observed upon treatment with increasing concentrations of ASEE. Significant differences are reported, with * indicating a *p*-value: 0.01 < *p* < 0.05, and ** indicating a *p*-value: 0.001 < *p* < 0.01.

Using WST-1 as a cell proliferation reagent, the percent proliferation of the AML cell lines, namely KG-1, Monomac-1, and U937, as well as the normal mesenchymal cells (MSCs), treated with *A. cherimola* seed ethanolic extract (ASEE), was calculated and the results show a dose- and time-dependent decrease in the proliferation of AML cell lines used. In fact, a significant time-dependent decrease in the proliferation of KG-1 cells is observed at all concentrations, with an IC_50_ of 57 µg/mL at 24 h and 20 µg/mL at 48 h (Figure 1A). A similar significant time-dependent decrease in proliferation is observed in U937 cells, with an IC_50_ of 100 µg/mL at 24 h and 50 µg/mL at 48 h (Figure 1B). In Monomac-1 cells, the reported IC_50_ is 107 µg/mL at 24 h and 85 µg/mL at 48 h (Figure 1C). On the other hand, only a slight decrease in MSCs proliferation is observed (a decrease in cell proliferation to 70%), indicating the selective inhibitory effect of the extract on AML cell lines and not MSCs (Figure 1D). Moreover, a statistically significant difference is observed when comparing the effect of various concentrations of ASEE on MSCs versus all AML cell lines at both 24 and 48 h, with *p*-values < 0.001 (Supplementary Figure S1).

### 2.2. A. cherimola Seeds Extract Induces an Increase in Sub-G1 Cells in AML Cell Lines

**Figure 2:**
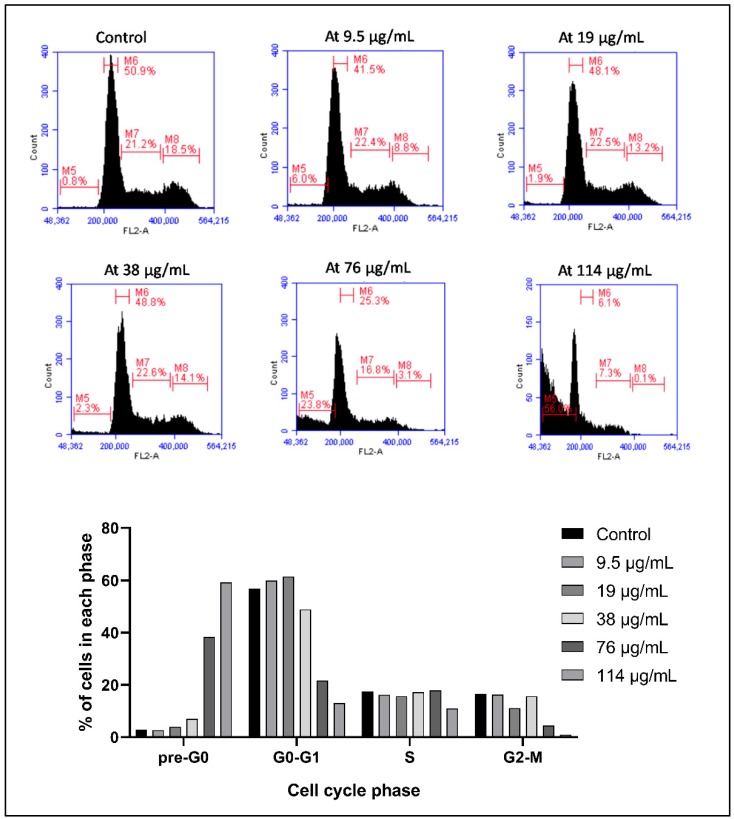
Cell cycle analysis of KG-1 treated with *Annona cherimola* seed ethanolic extract (ASEE) for 24 h. A dose-dependent increase in the sub-G1 phase and a decrease in the other cell cycle phases indicate cellular fragmentation in the cells upon treatment with increasing concentrations (9.5 µg/mL, 19 µg/mL, 38 µg/mL, 76 µg/mL, and 114 µg/mL) of ASEE for 24 h. M1 = sub-G1 phase, M2 = G0/G1 phase, M3 = S phase, and M4 = G2/M phase.

Using Propidium Iodide (PI) to assess the DNA content of cells, the distribution of the cells in the different cell cycle stages is elucidated and it shows a shift of the cells from the G0-G1, S, and G2-M stages to the sub-G1 stage, indicating cellular fragmentation. The distribution of KG-1 cells after 24 h follows a dose-dependent change between the control group, where only 2.8% of cells are in the sub-G1 stage, 56.8% in the G0-G1 stage, 17.4% in the S stage, and 16.5% in the G2-M stage, compared to the cells treated with 114 µg/mL, where 59.8% of cells are in the sub-G1 stage, 13.1% in the G0-G1 stage, 11% in the S stage, and 0.9% in the G2-M stage (Figure 2). 

**Figure 3:**
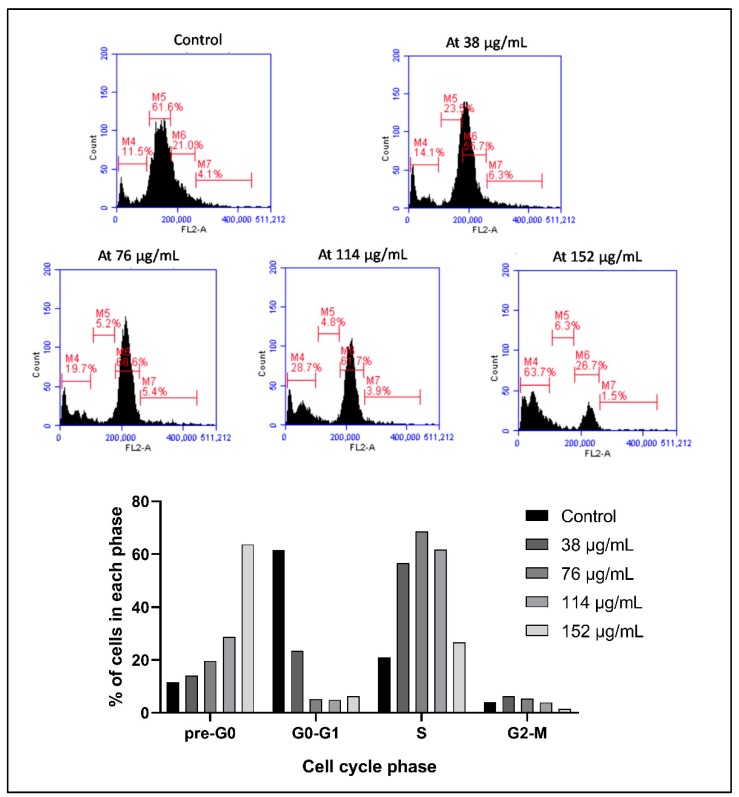
Cell cycle analysis of Monomac-1 treated with *Annona cherimola* seed ethanolic extract (ASEE) for 24 h. A dose-dependent increase in the sub-G1 phase and a decrease in the other cell cycle phases indicate cellular fragmentation of cells upon treatment with increasing concentrations (38 µg/mL, 76 µg/mL, 114 µg/mL, and 152 µg/mL) of ASEE for 24 h. M4 = sub-G1 phase, M5 = G0/G1 phase, M6 = S phase, and M7 = G2/M phase.

The same dose-dependent shift in the distribution of cells is observed in Monomac-1, whereby the percentage of cells in the sub-G1 stage increases from 11.5% in the controls to 63.7% upon treatment with 152 µg/mL ASEE for 24 h (Figure 3).

### 2.3. A. cherimola Seeds Extract Induces Apoptosis in AML Cell Lines

**Figure 4:**
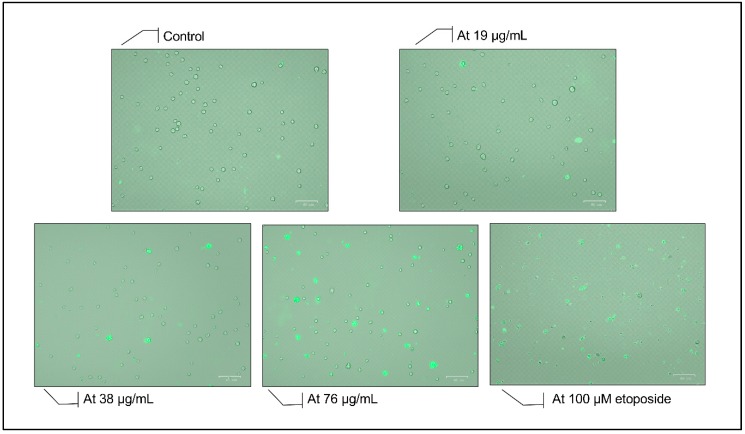
Annexin V staining of KG-1 cells treated for 24 h with increasing concentrations of *Annona cherimola* seed ethanolic extract (ASEE) and a positive control treated with etoposide. A gradual increase in the number of Annexin-positive cells is observed after 24 h between the control group and the concentrations before and after the IC50 (57 µg/mL), whereby most of the cells become positively stained at 76 µg/mL.

Using fluorescence microscopy, Annexin V binding to the cell membrane is detected, which is concomitant with the occurrence of apoptosis that is accompanied by flipping of the phosphatidyl serine moieties to the outer leaflet of the cell membrane. Figure 4 shows a gradual increase in the number of Annexin-positive cells after 24 h between the control cells and those treated with concentrations before and after the IC50 (57 µg/mL), whereby most of the cells become positively stained at 76 µg/mL, indicating an increase in apoptosis upon treatment with ASEE. The findings are compared to a positive control of cells treated with 100 μM etoposide to confirm their morphological changes.

**Figure 5:**
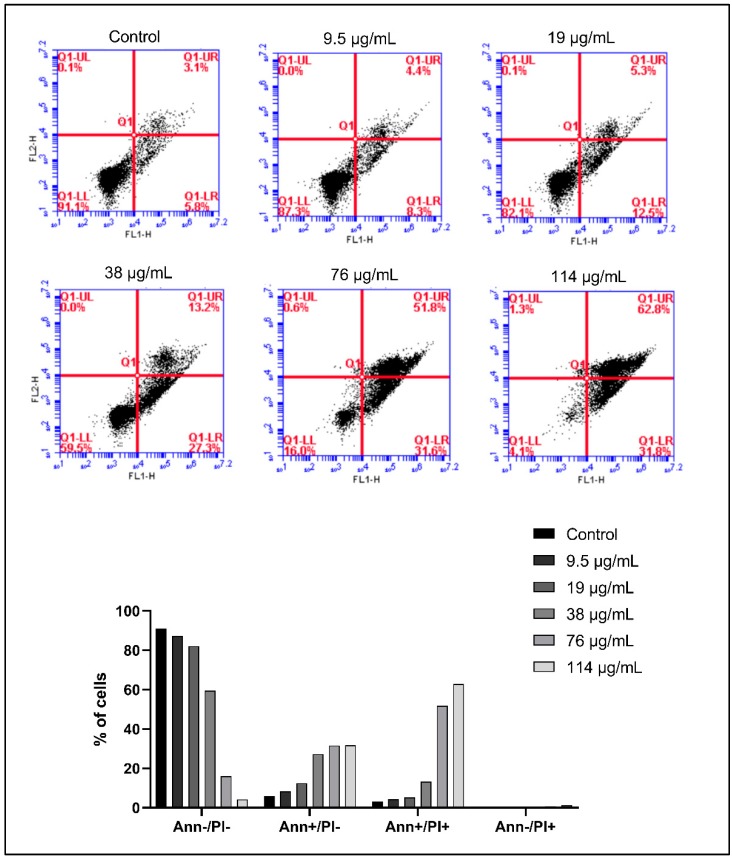
Annexin V/PI staining of KG-1 cells treated with increasing concentrations of *Annona cherimola* seed ethanolic extract (ASEE) for 24 h. A shift from double-negative staining to Annexin-positive/PI-negative staining double-positive staining and Annexin-negative/PI-positive staining is observed in KG-1 cells upon treatment with ASEE at the following concentrations: 9.5 µg/mL, 19 µg/mL, 38 µg/mL, 76 µg/mL, and 114 µg/mL.

**Figure 6:**
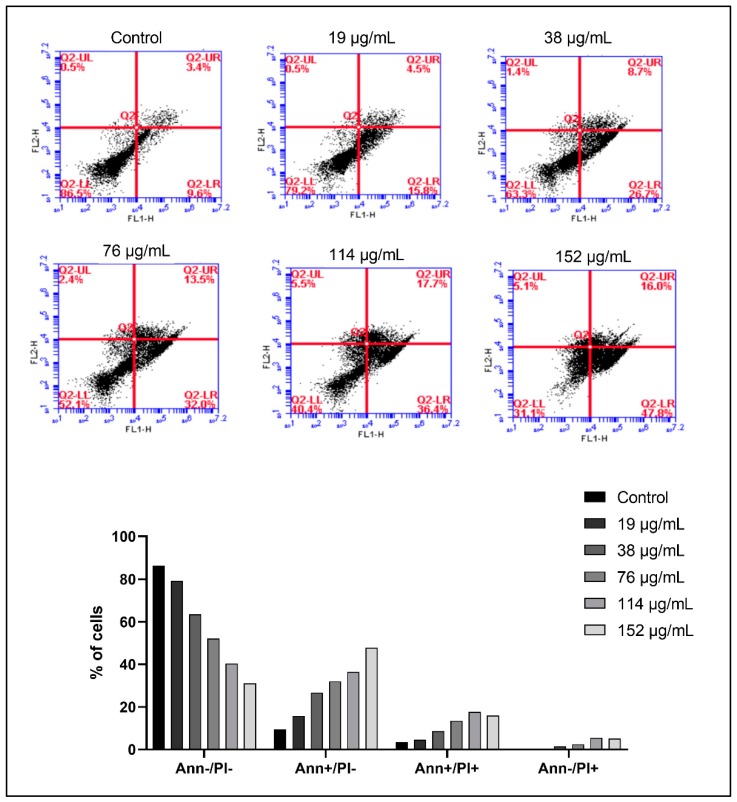
Annexin V/PI staining of Monomac-1 cells treated with increasing concentrations of *Annona cherimola* seed ethanolic extract (ASEE) for 24 h. A shift from double-negative staining to Annexin-positive/PI-negative staining, double-positive staining, and Annexin-negative/PI-positive staining is observed in Monomac-1 cells upon treatment with ASEE at the following concentrations: 19 µg/mL, 38 µg/mL, 76 µg/mL, 114 µg/mL, and 152 µg/mL.

To quantify the increase in apoptosis, Annexin V/PI dual staining was performed, followed by flow cytometry and analysis. Normal living cells exhibit negative staining to both PI and Annexin (lower left quadrant). However, early apoptotic cells exhibit negative staining to PI, but positive staining to Annexin (lower right quadrant), and late apoptotic cells exhibit positive staining to PI and Annexin (upper right quadrant). Necrotic cells exhibit positive staining to PI and negative staining to Annexin. In KG1 cells, we observe an increase in apoptosis upon treatment with 114 µg/mL of ASEE for 24 h, whereby 31.8% are in early apoptosis and 62.8% in late apoptosis, compared to 5.8% and 3.1% in untreated cells, respectively (Figure 5). A similar shift is observed in Monomac-1 cells upon treatment with 152 µg/mL of ASEE for 24 h, whereby 47.8% of cells are in early apoptosis, 16% are in late apoptosis, and only 31.1% remain normal and viable (Figure 6).

**Figure 7:**
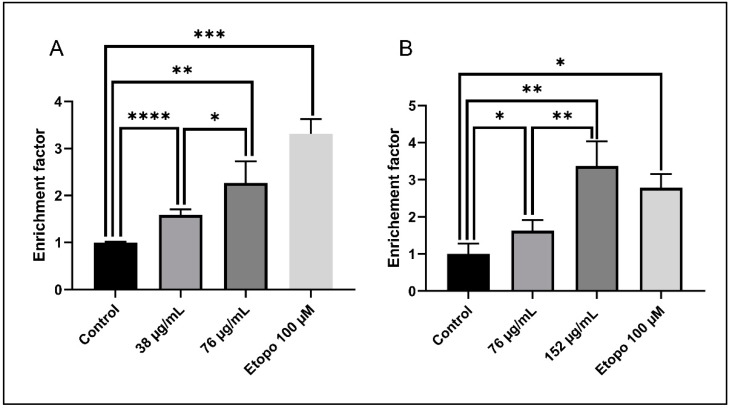
Cell Death ELISA on KG-1 cells (**A**) and Monomac-1 cells (**B**) treated with increasing concentrations of *Annona cherimola* seed ethanolic extract (ASEE) and a positive control treated with etoposide for 24 h. A significant dose-dependent increase in the enrichment factor is noted for KG-1 cells and Monomac-1 cells treated with two increasing concentrations of ASEE for 24 h. Significant differences are reported, with * indicating a *p*-value: 0.01 < *p* < 0.05, ** indicating a *p*-value: 0.001 < *p* < 0.01, *** indicating a *p*-value: 0.0001 < *p* < 0.001, and **** indicating a *p*-value: *p* < 0.0001.

To further confirm the increase in apoptosis, Cell Death ELISA was performed to quantify the levels of DNA fragmentation, which is a major hallmark of apoptosis. A significant dose-dependent increase in the enrichment factor is noted for both AML cell lines after 24 h. A 1.6-fold increase and 2.5-fold increase in the enrichment factor are observed in KG-1 cells treated with 38 µg/mL and 76 µg/mL, respectively (before and after the IC50 = 57 µg/mL) (Figure 7A). A similar effect is reported in Monomac-1 cells, whereby a 1.6-fold increase and 3.4-fold increase in the enrichment factor are observed upon treatment with 76 µg/mL and 152 µg/mL, respectively (before and after the IC50 = 107 µg/mL) (Figure 7B).

### 2.4. A. cherimola Seeds Extract Causes Upregulation of Pro-Apoptotic Proteins

**Figure 8:**
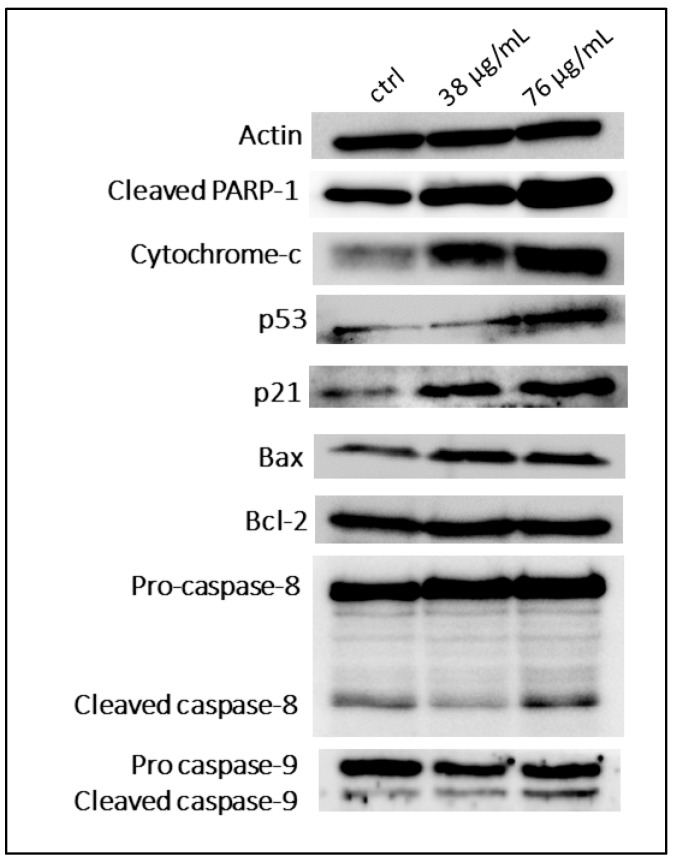
Western blot analysis of apoptosis-regulating proteins in KG-1 cells treated with *Annona cherimola* seed ethanolic extract (ASEE) for 24 h. Upregulation of pro-apoptotic proteins is observed between KG-1 control cells and KG-1 cells treated with 38 µg/mL or 76 µg/mL ASEE for 24 h.

**Figure 9:**
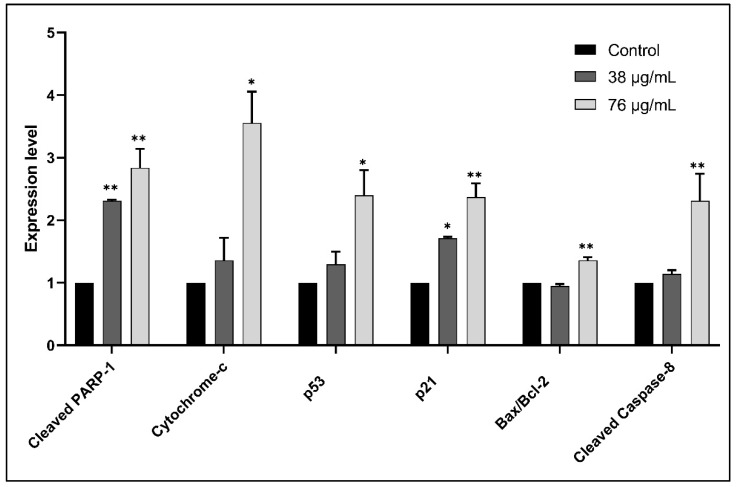
Quantification of the expression of apoptosis-regulating proteins in KG-1 cells treated with *Annona cherimola* seed ethanolic extract (ASEE) for 24 h. Significant upregulation of pro-apoptotic proteins is observed between KG-1 control cells and cells treated with 38 µg/mL or 76 µg/mL ASEE for 24 h. Upregulated proteins include cleaved PARP-1, cytochrome-c, cleaved caspase-9, the Bax/Bcl-2 ratio, p53, and cleaved caspase-8. Significant differences are reported, with * indicating a *p*-value: 0.0 < *p* < 0.05, and ** indicating a *p*-value: 0.001 < *p* < 0.01.

To identify the pathway by which ASEE inhibits proliferation and induces apoptosis, protein extraction from KG-1 cells was treated for 24 h with different concentrations of ASEE before and after the IC50 was performed, followed by SDS-PAGE and western blot for the quantification of key cell proliferation and apoptosis-regulating proteins. The results show an upregulation of key pro-apoptotic proteins such as p53, as well as cleaved PARP-1. The upregulation in Cytochrome-c indicates its release from the mitochondria. An increase in the agents of apoptosis, such as cleaved caspase-8 and cleaved caspase-9, is also detected, and an increase in the Bax to Bcl-2 ratio is noted as well (Figure 8 and Figure 9).

### 2.5. A. cherimola Ethanolic Seeds Extract Exhibits Antioxidant Properties

**Figure 10:**
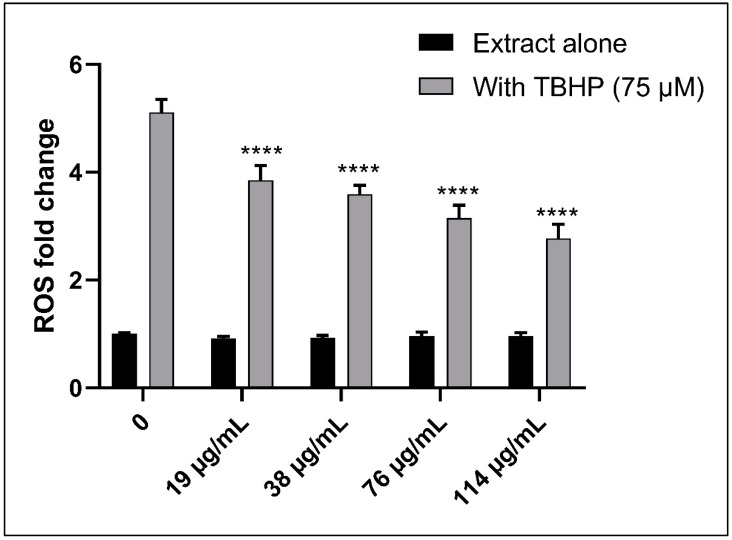
Fold change of ROS in DCFDA-stained KG-1 cells treated with either increasing concentrations of *Annona cherimola* seed ethanolic extract (ASEE) alone, or with a combination of 75μM Tert-Butyl Hydrogen Peroxide (TBHP) and increasing concentrations of ASEE. ROS levels remain constant in KG-1 cells upon treatment with ASEE alone, but they significantly decrease upon treatment with 75 μM TBHP along with increasing concentrations of ASEE. Significant differences are reported, with **** indicating a *p*-value: *p* < 0.0001.

Using the 2,7-dichlorofluorescin diacetate (DCFDA) Cellular Reactive Oxygen Species (ROS) Detection Assay kit, the ROS levels were measured, depending on the cleavage of the reduced non-fluorescent form of fluorescein to its fluorescent form by agents of oxidation in KG-1 cells. In fact, no significant change is observed in the ROS levels in the cells treated with ASEE alone, which indicates that the extract alone does not significantly induce or change the level of ROS in living cells. However, the addition of Tert-Butyl Hydrogen Peroxide (TBHP) (75 μM), which is known to be an ROS inducer, significantly increases the ROS level up to a 5.25-fold increase. Furthermore, adding 75μM of TBHP along with increasing concentrations of ASEE results in a gradual decrease in the ROS level induction by TBHP from a 5.25-fold increase in 75 μM TBHP only, down to a 2.98-fold increase in 75 μM TBHP with 114 µg/mL, which is a significant decrease when compared to the TBHP-only treated cells. This clearly indicates that ASEE has antioxidant properties since it can quench ROS induction by TBHP (Figure 10).

### 2.6. GC-MS Analysis of ASEE

**Figure 11:**
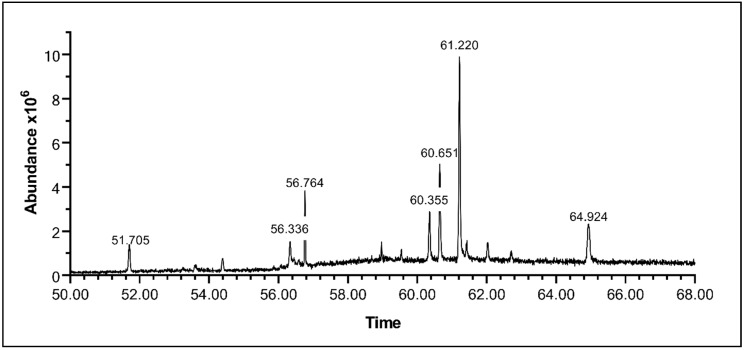
Chromatogram of *A. cherimola* Mill ethanolic seeds extract elucidated by GC/MS. Analysis reveals the presence of several compounds in varying amounts, as noted by the presence of several peaks at different retention times with varying areas under the peak.

To explore the composition of the extract, ASEE was analyzed using Gas Chromatography-Mass Spectrometry (GC-MS) in order to detect the presence of any small molecular weight, nonpolar, and volatile compounds possibly responsible for the anticancer properties of the extract. Several compounds were detected and most of them were identified (Figure 11; Table 1). The majority of identified compounds were phytosterols. Beta-Sitosterol is detected at Peak 6 (Retention Time = 61.2219 s), with an abundance of 37.8041%. Another abundant compound, Beta-Stigmasterol, is detected at Peak 5 (RT = 60.6504 s), with an abundance of 18.7066%. Other detected compounds are Dihydrobrassicasterol at Peak 4 (RT = 60.3532 s), with an abundance of 9.7107%, and Methylene DI-T-butylcresol at Peak 1 (RT = 51.7057 s), with an abundance of 6.5493%. Other unidentified compounds correspond to peaks at 56.3352 s, 56.7639 s, and 64.926 s, with abundances of 4.1458%, 10.2537%, and 12.83%, respectively.

## 3. Discussion

*A. cherimola* seeds are considered an important source of plant toxins which have been used as insecticides and linked to neurodegenerative diseases [3,4]. However, new research has shown their possible uses in cancer chemotherapy [14]. For that aim, an ethanolic extract of the seeds of *A. cherimola Mill* fruit was prepared due to the mild polarity index of ethanol [20], allowing for the extraction of many compounds, including the Annonaceous acetogenins known for their ethanol solubility [21]. The results reported in this study show that the extract has a dose-dependent anti-proliferative effect on all three AML cell lines used, namely KG-1, Monomac-1, and U937, with varying IC_50_ levels of 57 µg/mL, 107 µg/mL, and 100 µg/mL, respectively. These concentrations are potentially therapeutically effective considering that a previous study showed that the ethanolic extract from *Urtica membranacea* possessed potent anti-cancer effects at 750 μg/mL and 1500 μg/mL and these concentrations were efficiently correlated to mice breast cancer model treatment with no side effects [22].

The results also revealed a mild cytotoxic effect of the extract on MSCs, which was statistically lower than the cytotoxicity observed in AML cell lines. This further predicts the efficacy of the extract in targeting cancerous cells with minimal effects on normal cells, which is a crucial advantage of a chemotherapeutic drug with target selectivity [23]. The cytotoxic effect reported was also time-dependent, which could indicate the effectiveness of using the ASEE in combination with other drugs [24]. In this study, all remaining experiments were performed by exposing the cells to the extract for 24h in order to elucidate the mechanism of action of the extract in inhibiting cancer cell proliferation, even though therapeutic levels are best reached after 48 h incubation.

To explore the mechanism by which this previously considered toxic extract is exerting its anti-proliferative effects on the AML cell lines, KG-1 and Monomac-1 cells, which were more responsive to ASEE, were evaluated for cell cycle arrest and for any apoptotic changes. All experiments showed that the extract induced a dose-dependent increase in apoptosis levels in the cells, supported by an increase in cellular fragmentation, a flipping of the phosphatidylserine moieties to the outer leaflet of the cell membrane along with an increase in Annexin V staining of the cells, and an increase in DNA fragmentation. These apoptotic hallmarks, increasing between the control cells and the cells treated with the extract, are the result of the cellular activation of apoptotic pathways, by the mediation of several proteins [25]. Movement of the normally cytoplasm-facing phosphatidylserine to the outer leaflet of the plasma membrane increases the phagocytosis of apoptotic cells since phosphatidylserine acts as a recognition ligand for phagocytes [26]. 

Investigation of the apoptotic mechanisms activated in the cells treated with ASEE showed that both the intrinsic and extrinsic pathway of apoptosis are activated in a dose-dependent manner upon treatment and they coalesce to activate the execution pathway of apoptosis. The execution pathway is the final phase of apoptosis signaling where various intracellular mediators are activated, leading to the biochemical and morphological changes observed in apoptosis [27]. Among these substrates is Poly (ADP-ribose) Polymerase (PARP), which, upon cleavage, induces poly (ADP-ribosyl) ation of many nuclear proteins, which is an event crucial for apoptosis to proceed [28]. The reported increase in PARP cleavage further confirmed that the cytotoxicity exerted by ASEE is mediated by apoptosis.

As mentioned previously, ASEE treatment of AML cell lines also promoted apoptosis via an intrinsic pathway. This pathway is usually induced by stimuli that affect targets within the cell and cause mitochondrial-initiated events. Loss of mitochondrial transmembrane potential is usually followed by cytochrome-c release into the cytosol. Cytochrome-c then binds and activates pro-caspase-9, which leads to the upregulation of cleaved caspase-9. Other groups of proteins are released from the mitochondria and they are responsible for the DNA changes observed in apoptosis and detected by Cell Death ELISA [29]. This is where Bcl-2 family members come into play since they regulate mitochondrial membrane permeability, and among these members are the pro-apoptotic Bax and the anti-apoptotic Bcl-2, where the increased ratio observed upon treatment with the seed extract indicates a loss of mitochondrial membrane integrity, leading to the release of cytochrome-c and other related proteins.

Other proteins in the intrinsic pathway are effectors of p53-mediated apoptosis. The significant increase in p53 levels observed can be correlated to the upregulation of the apoptotic machinery by increasing the release of cytochrome-c from the mitochondria, and it can also lead to cell cycle arrest related to growth retardation. p53 is also speculated to regulate some members of the Bcl-2 family; however, the mechanism of inhibition is still not well-elucidated [30].

Moreover, ASEE also mediated apoptosis via the extrinsic pathway. The extrinsic or death receptor mediated pathway typically leads to the activation of caspase-8; our results revealed an upregulation of caspase-8, which was detected upon treatment with 76 µg/mL of ASEE. This suggests that the extrinsic pathway is only activated at high concentrations and that the intrinsic p53-dependent pathway plays a more crucial role in promoting the cell death of AML cells upon ASEE treatment.

Although ROS are known to activate apoptosis in the cell by causing the damage of subcellular components [31], antioxidant activity in plants was previously shown to be correlated with anticancer properties due to the presence of phytochemicals that induce apoptosis [32]. No changes in ROS levels were detected upon ASEE treatment, which indicated that the extract does not promote cell death by ROS induction. However, ASEE caused a significant decrease in ROS levels induced by TBHP treatment; this highlights the antioxidant activity of the extract being investigated. This has an important advantage since an extract with pro-apoptotic and antioxidant properties can potentiate the anticancer effects of chemotherapy and radiation therapy while protecting surrounding normal tissues vulnerable to side effects [33].

The analysis of the composition of the extract revealed the presence of β-sitosterol. β-sitosterol is a phytosterol previously studied for its anti-cancer effects; it was shown to have anti-proliferative and pro-apoptotic effects on U937 cells by selectively activating caspase-3 and increasing the Bax/Bcl-2 ratio, whereby the addition of z-DEVD-fmk (a caspase-3 specific inhibitor) or the overexpression of Bcl-2 significantly attenuated the apoptotic response [34]. Its ability to induce apoptosis was also reported in other cancer cells in vitro, such as human stomach cancer cells, human colon cancer cells, and human breast cancer cells [35,36,37].

Another detected compound was β-stigmasterol, which is a plant derivative of cholesterol. Even though it is less studied than β-sitosterol, it was shown to inhibit some cancer cells in vitro, like human laryngeal carcinoma cells [38]. As for Methylene DI-T-Butylcresol, it is a phenol derivative shown to induce autophagy in human embryonic kidney cells, potentiating the efficacy of belotecan, which is derivative from a chemotherapy drug [39].

Finally, acetogenins which have previously been reported in *A. cherimola* seeds [14] could not be quantified as the characterization of these polar and waxy molecules would require chromatographic purification followed by LCMS/MS and NMR analysis.

## 4. Conclusions

In conclusion, the ethanolic extract from the seeds of *Annona cherimola* Mill., a traditionally used plant seed with toxic properties, has been shown to exhibit a promising cytotoxic effect on AML cell lines, by inducing apoptosis through a p53-dependent mechanism and through the induction of both the intrinsic and extrinsic apoptotic pathways and decreasing the ROS levels.

## 5. Materials and Methods 

### 5.1. AML Cell Culture

The Acute Myeloid Leukemia cell lines Mono-Mac-1, U937, and KG-1 were obtained from the American Type Culture Collection. The cells were cultured in Roswell Park Memorial Institute medium (RPMI-1640, Sigma-Aldrich, St. Louis, MO, USA) enriched with 10% fetal bovine serum (FBS, Gibco^TM^, Dublin, Ireland) and antibiotics (100 U/mL penicillin and 100 µg/mL streptomycin from Pen-Strep, Lonza, Basel, Switzerland) in a humidified incubator in 5% CO_2_ at 37 °C. Before plating, cell viability was checked using the ZOE Fluorescent Cell Imager (Abcam, Cambridge, UK), along with Trypan Blue exclusion method.

### 5.2. Isolation and Culture of Mesenchymal Stem Cells from Rat Bone Marrow

MSCs were isolated from rat bone marrow according to a modified procedure. The single, 12-weeks old rat was provided by the animal facility at the Lebanese American University. It was maintained under optimal laboratory conditions and received food and water ad libidum, complying with the University’s Animal Care and Use Comity (ACUC) and the Guide for the Care and Use of Laboratory Animals [40,41]. The rat was sacrificed by CO_2_ asphyxiation and both hind legs were aseptically removed. Femoral and tibial bones were then isolated and washed with 70% ethanol and placed in sterile Phosphate Buffered Saline (PBS, Lonza, Basel, Switzerland) supplemented with 100 U/mL penicillin and 100 μg/mL streptomycin (Lonza). After removing the bone epiphyses with sterile scissors, bone marrows were flushed out using a needle filled with Dulbecco’s Modified Eagle Medium (DMEM, Sigma-Aldrich) supplemented with 10% Fetal Bovine Serum (Gibco^TM^) and 100 U/mL penicillin and 100 μg/mL streptomycin (Lonza). The cells collected were then incubated in vented flasks at 37 °C with 5% CO_2_. After 5 days of daily medium change, MSCs were identified by their spindle-shaped morphology, as observed using the ZOE Fluorescent Cell Imager [42,43].

### 5.3. Plant Material

*Annona cherimola* Mill fruits were taken from a tree in Awkar-Lebanon (34.4328° N, 35.9169° E, 90 meters above sea level), during January 2018 and were identified by the botanist Dr. Nisrine Machaka-Houri, according to the indications and characteristics described by Vanhove (2008) [44]. A voucher specimen was deposited in Beirut Arab University Herbarium (ID-RCED2019-362).

### 5.4. Preparation of the Crude Seed Extract

Fruit seeds were grinded and shaken with 80% ethanol at 200 rpm for 1 week. The extract was then filtered through a cheesecloth and spun at maximum speed to discard the pellet. Ethanol was evaporated using a rotary evaporator. The dried extract was weighed and then dissolved in Dimethyl sulfoxide (DMSO) and diluted with RPMI to obtain a crude extract with a final concentration of 3800 µg/mL. When applied on the cell lines, the DMSO level maximally reached 0.4% at 150 µg/mL. 

### 5.5. Cytotoxicity Assay

AML cells and MSCs were seeded in 96-well plates at a density of 0.5 × 10^5^ cells/well and incubated overnight. Triplicates of wells were treated with increasing concentrations of *A. cherimola* seeds ethanolic extract (ASEE) (9.5–152 μg/mL). Control cells were treated with RPMI media. The plates were incubated for 24 h or 48 h before the addition of WST-1 cell viability reagent (Roche, Basel, Switzerland), according to the manufacturer’s guidelines. Absorbance at 450 nm was measured by spectrophotometry using a Multiskan^TM^ FC Microplate Photometer to detect metabolically active cells and the percentage proliferation was calculated.

### 5.6. Cell Cycle Analysis Using PI Staining

AML cells were seeded in 6-well plates at a density of 1 × 10^5^ cells/well and incubated overnight. After incubation for 24 h or 48 h with increasing concentrations of the ASEE (9.5–152 μg/mL, control cells were treated with RPMI media), the cells were fixed with ice-cold absolute ethanol for fixation and stained with PI (Abcam, Cambridge, UK). The DNA content was assessed using the Accuri C6 flow cytometer. The distribution of cells in each cell cycle phase was determined by assessing the DNA content: sub-G0/G1 phase cells (Pre-G or dead cells) have <2n, G0/G1 phase cells have 2n, S phase cells have between 2n and 4n, and G2/M phase cells have 4n.

### 5.7. Apoptosis Detection Using Annexin V Staining by Fluorescence Microscopy

AML cells were seeded in 24-well plates at a density of 1 × 10^5^ cells/well and incubated overnight. After incubation for 24 h or 48 h with increasing concentrations of the ASEE (19–76 μg/mL, control cells were treated with RPMI media), cells were stained with Annexin using the Annexin V–Fluorescein Isothiocyanate (FITC) Apoptosis Detection Kit (Abcam, Cambridge, UK). The cells were visualized under the ZOE Fluorescent Cell Imager using bright-field conditions and the filter set was then for FITC before merging the images.

### 5.8. Apoptosis Quantification by Annexin V/PI Staining

AML cells were seeded in a 6-well plate at a density of 2 × 10^5^ cells/well and incubated overnight. After incubation for 24 h or 48 h with increasing concentrations of the ASEE (9.5–114 μg/mL, control cells were treated with RPMI media), cells were stained with Annexin V and PI (Annexin V–FITC Apoptosis Detection Kit, Abcam, Cambridge, UK) and immediately analyzed using the Accuri C6 flow cytometer. Annexin V binds in a Ca^2+^-dependent manner to the exposed charged head groups of phosphatidylserine, which is translocated to the outer leaflet of the cell membrane upon apoptosis. The cell membrane integrity excludes PI in viable and apoptotic cells, but not in necrotic cells. Therefore, dual parameter FACS (Fluorescence-activated cell sorting) analysis allows for the discrimination between viable, apoptotic, and necrotic cells.

### 5.9. Apoptosis Detection Using Cell Death ELISA

AML cells were seeded in a 12-well plate at a density of 0.25 × 10^5^ cells/well and incubated overnight. Duplicates of wells treated with two increasing concentrations of ASEE (38–76 μg/mL for KG-1 and 76–152 μg/mL for Monomac-1) were plated for 24 h. Control cells were treated with RPMI media. Positive control wells of cells were treated with 100 μM (58.85 µg/mL) of etoposide (Abcam, Cambridge, UK). Using the Cell Death ELISA kit (Roche, Basel, Switzerland), cells were extracted and lysed with incubation buffer before the isolation of fragmented cytosolic DNA. Microplate wells were coated with anti-histone antibodies. Extracted DNA was then incubated in the wells, which were washed before adding anti-DNA antibodies linked to an enzyme, and then washed again before adding an ABTS (2,2′-azino-bis(3-ethylbenzothiazoline-6-sulphonic acid) colorimetric substrate. Absorbance at 405 nm (and 492 nm as a background) was measured by spectrophotometry using the Multiskan^TM^ FC Microplate Photometer and DNA fragmentation enrichment factor (absorbance of treated cells/absorbance of non-treated cells) was calculated based on manufacturers’ instructions.

### 5.10. Western Blot

KG-1 cells were plated in a 6-well plate at a density of 10^6^ cells/mL before treatment with two increasing concentrations of ASEE for 24 h (38 and 76 μg/mL). Control cells were treated with RPMI media. Total proteins were extracted using the Q-proteome Mammalian Protein kit (Qiagen, Hilden, Germany) and quantified using the DC (Detergent Compatible) protein assay (Bio-Rad). Proteins were separated by SDS-PAGE; transferred to PVDF (Polyvinylidene fluoride) membranes which were blocked with 5% skimmed milk; and then incubated with primary antibodies anti-β-actin (Santa Cruz Biotechnology, Dallas, TX, USA), anti-Cytochrome-c, anti-cleaved PARP-1, anti-Bax, anti-Bcl2, and anti-caspase-9 (Abcam, Cambridge, UK), anti-p53, and anti-caspase-8 (Elabscience, Houston, TX, USA) at the manufacturer’s recommended concentrations. After washing and incubation with a secondary antibody (Bio-Rad, Hercules, CA, USA), membranes were washed and image development was done using the Clarity™ Western ECL Substrate (Abcam, Cambridge, UK) on the ChemiDoc machine (BioRad, Hercules, CA, USA). Blot bands were quantified using the ImageJ computer program to calculate the relative expression of proteins.

### 5.11. Reactive Oxygen Species Detection

The DCFDA Cellular ROS Detection Assay kit (Abcam, Cambridge, UK) was used to detect the levels of Reactive Oxygen Species (ROS) in the cells. KG-1 cells were incubated with the cell-permeant 2′,7′-dichlorodihydrofluorescein diacetate (H_2_DCFDA), and then plated in duplicates in a 96-well plate and treated with either increasing concentrations of ASEE (19–114 μg/mL), or with a combination of 75 μM of Tert-Butyl Hydrogen Peroxide (TBHP) with increasing concentrations of the ASEE (control cells were treated with RPMI media). TBHP is a potent ROS inducer inside the cells, so it was used as a positive control. H_2_DCFDA (the chemically reduced form of fluorescein) acts as an indicator for ROS in cells upon its oxidative conversion to the highly fluorescent 2′,7′-dichlorofluorescein (DCF), which was quantified by fluorescent spectroscopy on the Varioskan™ LUX multimode microplate reader (Thermo Fisher Scientific, Waltham, MA, USA).

### 5.12. Gas Chromatography Mass Spectrometry Analysis of the Ethanolic Extract of A. Cherimola Seeds

The extract was analyzed using GCMS. The carrier gas was helium with splitless injection and the flow rate of 1.2 mL/min was applied. The temperature program was 2.0 min at 70 °C, from 70 to 130 °C at 8 °C/min and hold for 5 min, from 130 to 180 °C at 2 °C/min and hold for 10 min, from 180 to 220 °C at 15 °C/min and hold for 2 min, and then from 220 to 280 °C at 15 °C/min and hold for 22 min. Preliminary identification of the various compounds was performed by comparing their mass spectra with the literature (NIST11 and Wiley9 mass spectral databases). The percentage composition was computed from GC peak areas.

### 5.13. Statistical Analysis

All the experiments were repeated three times. Statistical analyses were performed using GraphPad Prism 8 (San Diego, CA, USA). The error bars were given as the mean ± SEM and the *p*-values were calculated by *t*-tests or two-way ANOVA, depending on the experiment. Significant differences were reported, with * indicating a *p*-value: 0.01 < *p* < 0.05, ** indicating a *p*-value: 0.001 < *p* < 0.01, *** indicating a *p*-value: 0.0001 < *p* < 0.001, and **** indicating a *p*-value: *p* < 0.0001.

## Figures and Tables

**Table 1:** Table showing the composition of the *A. cherimola* ethanolic seeds extract, as elucidated by GC-MS.

Peak	Retention Time	Compound	% of the Extract
1	51.7057	Methylene DI-T-butylcresol	6.5493
2	56.3352	Unidentified A	4.1418
3	56.7639	Unidentified B	10.2537
4	60.3532	Dihydrobrassicasterol	9.7107
5	60.6504	Beta-Stigmasterol	18.7066
6	61.2219	Beta-Sitosterol	37.8041
7	64.926	Unidentified C	12.83

## Supplementary Materials

The following are available online at https://www.mdpi.com/2072-6651/11/9/506/s1: Figure S1: Comparison of the proliferation reduction induced by various concentrations of ASEE in MSCs versus AML cell lines (KG-1, Monomac-1 and U937) at 24 h (**a**) and 48 h (**b**). **** indicates a *p*-value < 0.001.
