# Supplementary material for: Annona cherimola Seed Extract Activates Extrinsic and Intrinsic Apoptotic Pathways in Leukemic Cells
Source: Toxins (Basel). 2019 Aug 30;11(9):506. doi: 10.3390/toxins11090506 (PMC6784061; doi:10.3390/toxins11090506)
Supplement: Supplementary file 1 [file toxins-11-00506-s001.pdf]

Supplementary Materials: *Annona cherimola* Seed Extract Activates Extrinsic and Intrinsic Apoptotic Pathways in Leukemic Cells

Tony Haykal, Peter Nasr, Mohammad H. Hodroj, Robin I. Taleb, Rita Sarkis, Marvy Nadine El. Moujabber and Sandra Rizk

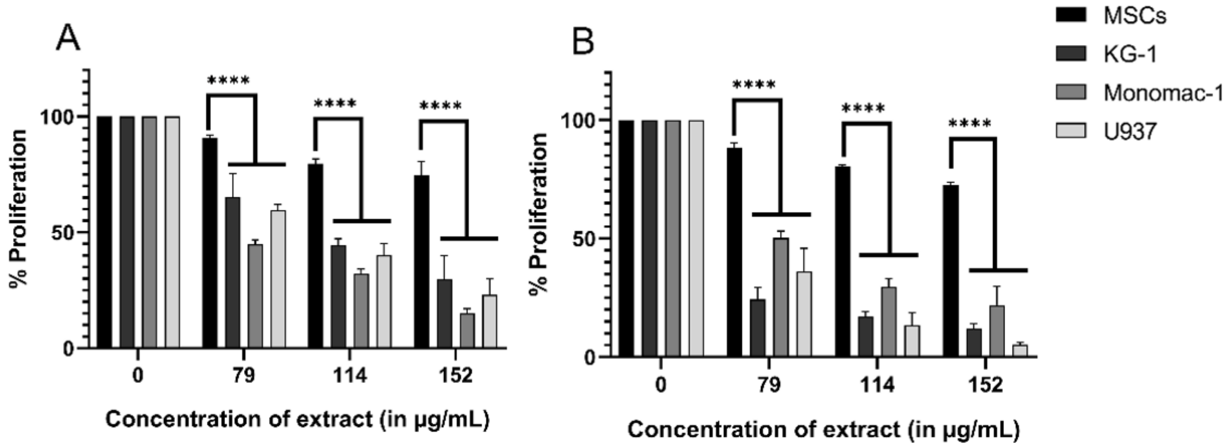

Figure S1. Comparison of the proliferation reduction induced by various concentrations of *Annona cherimola* seed ethanolic extract (ASEE) in normal mesenchymal cells (MSCs) versus acute myeloid leukemia (AML) cell lines (KG-1, Monomac-1, and U937) at 24 h (**a**) and 48 h (**b**). **** indicates a p-value < 0.001.
